# Verifying 4D gated radiotherapy using time-integrated electronic portal imaging: a phantom and clinical study

**DOI:** 10.1186/1748-717X-2-32

**Published:** 2007-08-30

**Authors:** John R van Sörnsen de Koste, Johan P Cuijpers, Frank GM de Geest, Frank J Lagerwaard, Ben J Slotman, Suresh Senan

**Affiliations:** 1 Department of Radiation Oncology, VU University medical center, Amsterdam, The Netherlands

## Abstract

**Background:**

Respiration-gated radiotherapy (RGRT) can decrease treatment toxicity by allowing for smaller treatment volumes for mobile tumors. RGRT is commonly performed using external surrogates of tumor motion. We describe the use of time-integrated electronic portal imaging (TI-EPI) to verify the position of internal structures during RGRT delivery

**Methods:**

TI-EPI portals were generated by continuously collecting exit dose data (aSi500 EPID, Portal vision, Varian Medical Systems) when a respiratory motion phantom was irradiated during expiration, inspiration and free breathing phases. RGRT was delivered using the Varian RPM system, and grey value profile plots over a fixed trajectory were used to study object positions. Time-related positional information was derived by subtracting grey values from TI-EPI portals sharing the pixel matrix. TI-EPI portals were also collected in 2 patients undergoing RPM-triggered RGRT for a lung and hepatic tumor (with fiducial markers), and corresponding planning 4-dimensional CT (4DCT) scans were analyzed for motion amplitude.

**Results:**

Integral grey values of phantom TI-EPI portals correlated well with mean object position in all respiratory phases. Cranio-caudal motion of internal structures ranged from 17.5–20.0 mm on planning 4DCT scans. TI-EPI of bronchial images reproduced with a mean value of 5.3 mm (1 SD 3.0 mm) located cranial to planned position. Mean hepatic fiducial markers reproduced with 3.2 mm (SD 2.2 mm) caudal to planned position. After bony alignment to exclude set-up errors, mean displacement in the two structures was 2.8 mm and 1.4 mm, respectively, and corresponding reproducibility in anatomy improved to 1.6 mm (1 SD).

**Conclusion:**

TI-EPI appears to be a promising method for verifying delivery of RGRT. The RPM system was a good indirect surrogate of internal anatomy, but use of TI-EPI allowed for a direct link between anatomy and breathing patterns.

## Background

The AAPM Task Group Report 76 recommended that respiratory motion management technology be considered when tumor motion exceeds 5 mm [1]. The least intrusive and most patient friendly of the available methods for motion management appears to be respiratory gating, and planning studies indicate that reduction in radiation toxicity can be reduced using this approach [2-4]. Restricting the period for treatment delivery to either end-expiration or end-inspiration will allow for smaller internal target volumes (ITV) to be treated during RGRT [5,6]. The overall accuracy of RGRT delivery is dependent on the accuracy of daily patient setup [7-9], and on the reproducibility of tumor position during both the daily treatments [10,11] and the overall treatment course [12,13]. Reproducibility of tumor position during RGRT is of concern [14] and pre-treatment verification is important [1]. As lung tumors are often not clearly visualized on fluoroscopy [12], fiducial markers implanted in or nearby the lung tumor region have been used [15,16]. Drawbacks of using fiducial markers include the risk of pneumothorax during transthoracic placement [17] and high drop-out rates when markers are inserted via a bronchoscope [18].

Gating systems that use motion signals from the abdominal wall as a surrogate for internal tumor motion can be unreliable if variations in correlation and phase shifts arise between the surrogate and internal structures [18-21]. Other approaches for improving the reproducibility of tumor position include spirometer-based active breathing control devices [22,23], and with audio or audio-visual respiratory coaching [24-27]. Even when such measures are taken, it is desirable to perform pre-treatment imaging to verify gating accuracy. In this report, we describe a time-integrated electronic portal imaging (TI-EPI) procedure that can be used to verify RGRT.

## Methods

### Electric portal imaging device (EPID) and Time-integrated EPI acquisition (TI-EPI)

An amorphous silicon-based EPID system (aSi500, Varian Medical Systems) mounted on a Varian 2300 C/D Linac (6–15 MV) equipped with a 120 dynamic MLC (Varian Medical Systems) was used for all studies. The EPID system consists of an image detection unit (IDU) featuring detector and accessory electronics, an image acquisition unit containing drive, acquisition electronics and interfacing hardware, and a dedicated workstation for off-line image review (Portal Vision 6.5, Varian Medical systems). The IDU matrix consist 512 × 384 pixels (pixel size: 0.78 × 0.78 mm) enabling a 40 × 30 cm^2 ^sensitive area at 145 cm source detector distance, i.e. 27.5 × 20.7 cm^2 ^with typical 100 cm isocenter-based radiation techniques. The use of this system for retrospectively verifying IMRT dose delivery was previously reported [28,29]. We acquired TI-EPIs in the dosimetric acquisition mode, which enables the continuous buffering of beam dose data exiting the patient. As portal dose verification in the previous work derived the integral exit dose, we postulated that the corresponding integral grey values comprising the EPI would reveal time-related positional information for mobile objects

### Phantom study

**Figure 1:**
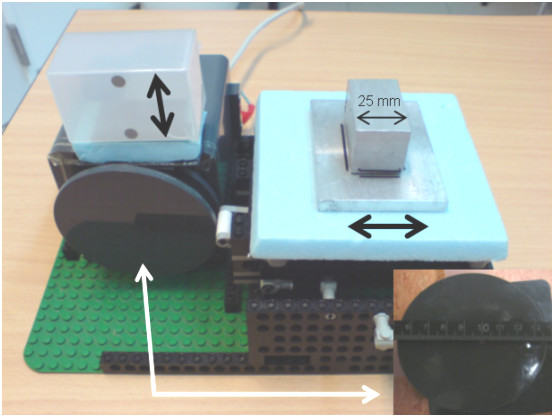
View of respiratory motion phantom used, with a detailed view of the cam shown (below, right).

A standard respiratory motion phantom tool (GE Varian 4D solutions) was modified with an AC/DC device that allowed setting of constant full cam rotation time (1R) of 4 sec, which reflects the mean breathing cycle duration in patients [30]. For this study, a second identical cam was added that had a 90 degree clock-wise rotation which allowed collection of TI-EPI data from an aluminum block placed on a platform with mobility direction perpendicular (i.e. horizontal) to the RPM marker (Figure 1). With a full (0–2π) rotation of the cam and 4.0 sec 1R cam rotation time, both the RPM marker and the phantom block have a mean sinusoidal motion of 5.5 mm/sec, synchronized in phase and amplitude of motion. Due to the design of the cam shape, objects move faster and slower during the simulated respiratory cycles. For example, a 9 mm motion amplitude is obtained during 1 1/2 π – 0π – 1/2 π rotation periods, while a 2 mm motion amplitude is seen during 1/2 π – 1π – 1 1/2 π rotation periods, with 0π and 1π, respectively, corresponding to the maximum inspiration and expiration positions. The duration of end-respiration periods presents only about 30% of the full duration cycle time.

In order to study the absolute grey value comparisons of TI-EPI images, phantom measurements were performed using portal acquisition settings used in clinical gating delivery. TI-EPI acquisition parameters were 6 MV (photon energy), 100 MU (Monitor Units, 1 MU = 1 cGy) dose delivery, in 600 MU/min dose rate. TI-EPI acquisitions were performed in the following settings: (i) an immobile (aluminum) block at end-inspiration and end-expiration (ii) the same block moving during simulated RGRT scenarios in end-expiration and end-inspiration, and (iii) continuous acquisition mode during all block movements. The latter was repeated for double exposure time (200 MU).

### Analyses of phantom TI-EPI data

The 16-bit format of TI-EPI portals was converted to 8-bit to allow for a 0–255 grey-value display. Imaged object positions on TI-EPI were derived by grey-value profile plots generated from an 8 cm fixed trajectory in line with object motion. Temporal object information was derived using a pixel-based grey-value subtraction means. A public domain software package, ImageJ [30] was used to generate the profiles and to re-format the portal data. Both phantom motion data in ASCI file format and the profile data were analyzed in Excel software (Microsoft Redmond, WA).

### Four dimensional CT scans (4DCT) of patients

Patients suitable for RGRT undergo customized audio-coaching in order to ensure reproducible breathing at time of 4DCT imaging and during RGRT delivery. Our 4DCT imaging procedure has been described previously [31,32]. Briefly, patients are scanned in supine position on a LightSpeed 16-slice CT scanner (General Electric Company, Waukesha, WI). During 4DCT acquisition, the respiratory pattern of the patient is logged using the Varian Real-time Position Management (RPM) respiratory gating system (Varian Medical Systems, Palo Alto, CA), and same system is used at the treatment unit to (i) verify reproducible patient breathing patterns and (ii) trigger beam on/off signals when a stable breathing pattern is observed and selected gating widows are in range. The RPM system uses two infrared light-reflecting markers attached on a plastic box placed midway between umbilicus and xiphoïd, and the box is secured in the marked position with adhesive tape. The reflective markers are illuminated by infrared-emitting diodes surrounding a CCD camera located at foot end of the scanner cradle. Vertical motion of these markers is captured by the camera at a frequency of 25 frames per second, and RPM software calculates the respiratory phase on the basis of signal processing of the observed amplitude. The RPM file and CT images are loaded to an Advantage Workstation 4.1 (General Electric Company, Waukesha, WI), where the Advantage 4D CT application assigns a specific respiratory phase to each image, and phase-related images are then saved in one of ten relative respiratory phase bins. The resorted CT phase bin '0%' typically defines the extreme end-inspiration position, and extreme end-expiration is generally represented in either the 50% or 60% phase. The phase-sorted data sets were reviewed in an Advantage 4D browser program.

### Patients data generated during RGRT

Patient 1 was treated with concurrent chemo-radiotherapy for stage III lung cancer in the right lower lobe. RGRT at three end-inspiration phases was performed to a dose of 60 Gy in 30 once-daily fractions delivered using 6 MV photons. Patient 2 presented with a recurrent solitary liver metastasis that was treated to 60 Gy in once-daily fractions of 3 Gy, during three end-expiratory phases using 15 MV photons. In order to account for variations in respiration that may persist despite audio-coached respiration, we expand the ITV symmetrically in the cranio-caudal direction by a 5 mm margin, followed by the addition of a symmetric three-dimensional margin of 10 mm to account for both microscopic extension and patient setup errors.

**Figure 2:**
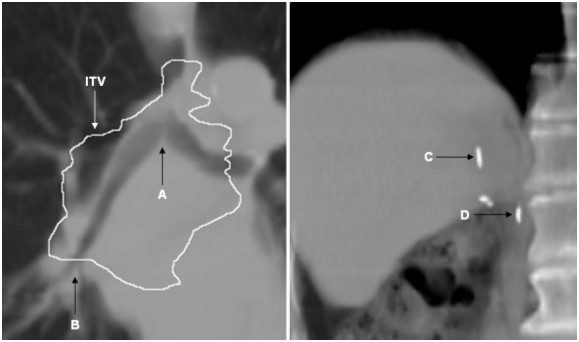
Tumor surrogates imaged in patient 1 (left) were the carina bifurcation (A), proximal bronchus bifurcation (B) and the internal target volume (ITV) contour is superimposed. Cranial (C) and caudal clips (D) were surrogates for patient 2.

All TI-EPI portals were registered in ImageJ to the reference portal derived from digitally reconstructed radiographs (DRR) from the average-intensity projection of three CT data sets used to define the gate. In order to exclude inter-observer variations, one observer performed all bony image registrations and identified visible anatomic structures on both TI-EPI and DRR. In patient 1, the right bronchial tree extending from the main carinal to a proximal bronchus junction was digitally marked on all images (fig 2). For patient 2, two surgical clips located in the tumor-bed were used to compare daily TI-EPIs with the DRR (fig 2). The maximum cranial-caudal mobility of selected internal structures were derived from 4DCT scans. Image analysis was performed using ImageJ and data were analyzed in Microsoft Excel software.

## Results

### Phantom motion

ASCI file records of phantom motion showed the cam rotation time to be constant at 4.1 sec/R, with peak-to-peak motion amplitude of 11 mm. The duration of the end-inspiration and the end-expiration gating windows that spanned three successive respiratory phases was 1.2 seconds. Maximum residual marker motion at the end-inspiration window was 4.0 mm, but was minimal (< 1 mm) in end-expiration.

### TI-EPI acquisitions with phantom

With a 10 sec (100 MU) uninterrupted exposure at a dose rate of 600 MU/min, TI-EPI collected approximately 104 frames with a negligible sync delay time for the first frame. The number of collected frames is proportional to time and this is information is available after completion of TI-EPI acquisition. For TI-EPI acquisition of a phase-gated treatment field, only a subset number of the total frames will contain data, with the remainder being blanks during 'beam-off' periods.

**Figure 3:**
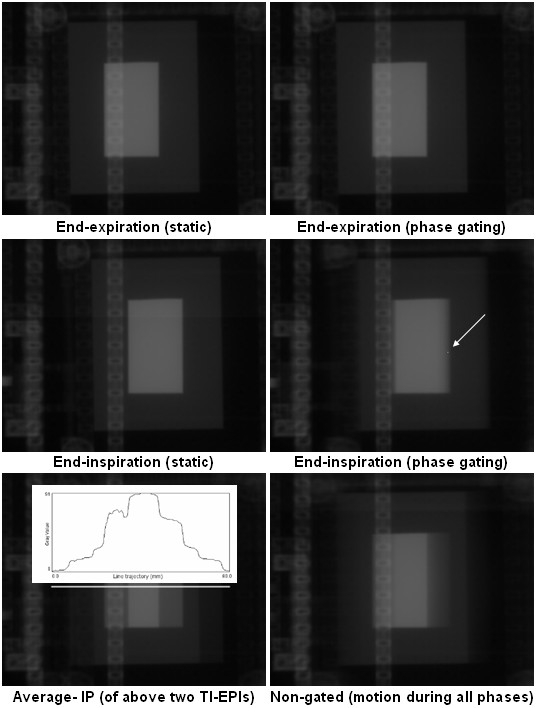
TI-EPI portals of the phantom imaged in static and moving (gated) mode in both inspiration and expiration. The white arrow on the "End-inspiration (phase gating)" portal points to a pixel location (white dot) of which temporal block information was derived. The lowermost panels shown the merged TI-EPI portals at end-inspiration and end-expiration (bottom left), and the corresponding non-gated image is also shown (bottom right). The bottom left portal shows the profile plot of measured grey values along an 8 cm white line.

**Figure 4:**
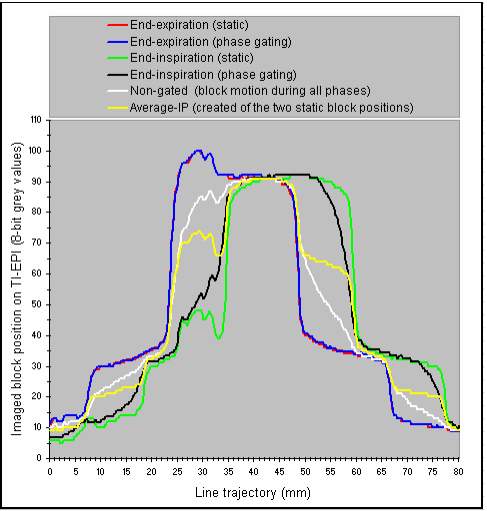
Experiment profile plots of the TI-EPI derived from a fixed 8 cm line trajectory.

**Figure 5:**
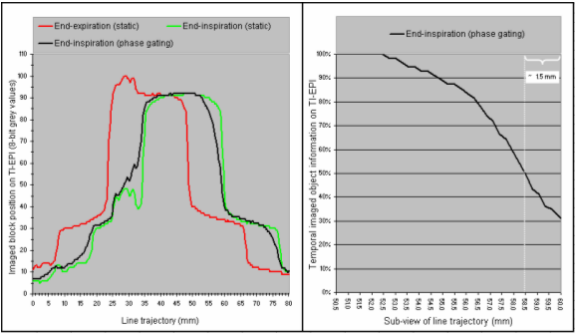
The profile plot of end-inspiration phase gating and the profile plots that were used to derive the background value of pixels are shown (left panel), and the temporal information derived from TI-EPI portal of end-inspiration phase gating shows right. The 'temporal' information, i.e. frequency of imaging of the block at pixels of the "line trajectory" during end-inspiration TI-EPI acquisition, shows in a ratio (grey value of mobile object/grey value of static object). For this ratio the background grey-value of pixels was derived by subtracting the pixel grey values measured by the "red profile" from those measured by "green profile" (left panel).

The main findings of the phantom experiment were as follows. Firstly, grey value profiles along the line trajectory at simulated 'end-expiration gating' were almost identical to TI-EPI in static end-expiration position, which indicates residual block motion of < 1 mm (Figures 3, 4). Secondly, TI-EPI during gating at end-inspiration phase showed blurring at both sides of the block, and analysis showed that the block remained at the location of the white pixel for 50% of the time (Figures 3, 4). Relative to the maximum end-inspiration position, the location of the marker (white) pixel was shifted 1.5 mm in the direction of expiration (Figure 5). Thirdly, non-gated TI-EPI acquisition during motion showed significant blurring and revealed the overall mean block position (Figures 3, 4). This image differed from an 'average intensity' projection created by merging portals of imaged static block positions acquired at end-inspiration and end-expiration, where the imaged time components of the blocks are per definition equal. TI-EPI during free breathing results in more data frames captured at end-expiration as the block moves much slower during these periods. Finally, TI-EPI portals acquired during non-gated movement for 10 seconds, during which 2.5 breathing cycles were captured, were similar to images acquired for a 20 sec acquisition (5 breathing cycles). This indicates that image quality and information was not affected by shorter periods of imaging.

### TI-EPI acquisitions of patient data

As patient setup is routinely measured using an EPI protocol, no TI-EPIs were acquired during the first three treatment fractions. The motion of intra-thoracic structures and marker clips of both patients in all phases of the 4DCT, and in the selected gating phases, are summarized in Tables 1 and 2.

**Table 1:** Motion of intra-thoracic structures in patient1 (i) during audio-coached 4DCT scan, (ii) in the gating phases of 4DCT scan, and (iii) TI-EPI during phase end-inspiration RGRT.

**Patient 1**	4DCT (all phases)				4DCT gated (three phases)	
	
	X (Max motion)	**Y **(Max motion)	X (1 SD motion)	**Y **(1 SD motion)	X (Max motion)	**Y **(Max motion)
Carina (bifurcation)	2.8 mm	10.0 mm	1.2 mm	3.8 mm	1.8 mm	2.5 mm
Proximal bronchus (bifurcation)	3.7 mm	17.5 mm	1.3 mm	5.7 mm	1.9 mm	5.0 mm

End-inspiration gating (26 treatments)	Gating reproducibility (excluding patient set-up errors)					
			
	X (Mean error)	**Y **(Mean error)	X (1 SD motion)	**Y **(1 SD motion)		
		
Bronchial tree	0.7 mm to lateral	2.8 mm to cranial	0.7 mm	1.6 mm		

**Table 2:** Motion of fiducial markers in patient 2 (i) during audio-coached 4DCT scan, (ii) in the gating phases of 4DCT scan, and (iii) TI-EPI during phase end-expiration RGRT.

**Patient 2**	4DCT (all phases)				4DCT gated (three phases)	
	
	X (Max motion)	**Y **(Max motion)	X (1 SD motion)	**Y **(1 SD motion)	X (Max motion)	**Y **(Max motion)
Cranial clip	1.0 mm	20.0 mm	0.3 mm	8.3 mm	0.0 mm	2.4 mm
Caudal clip	2.0 mm	18.6 mm	0.7 mm	6.7 mm	0.0 mm	2.2 mm

End-expiration gating (17 treatments)	Gating reproducibility (excluding patient set-up errors)					
			
	X (Mean error)	**Y **(Mean error)	X (1 SD motion)	**Y **(1 SD motion)		
		
Cranial clip	1.0 mm to lateral	0.4 mm to caudal	0.6 mm	1.5 mm		
Caudal clip	1.0 mm to lateral	1.4 mm to caudal	0.8 mm	1.4 mm		

In patient 1, the 4DCT phases selected for RGRT revealed that residual motion of the carina bifurcation decreased from 10.0 mm to 2.5 mm, and motion of the proximal bronchus bifurcation reduced from 17.5 mm to 5.0 mm. Data acquired from TI-EPI during 26 fractions showed the inter-fraction variation in position of the bronchial tree to be 1.6 mm (1 SD). The systematic 'gate error' in bronchus position was 2.8 mm (cranial displacement), but the extra cranio-caudal ITV margin of 5 mm ensured target coverage.

**Figure 6:**
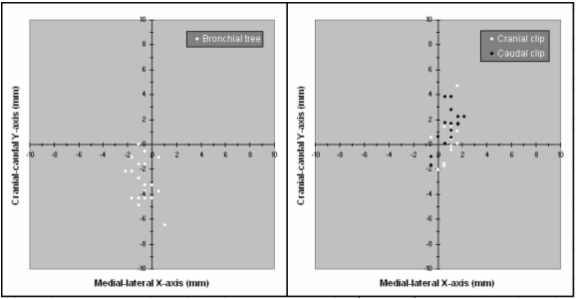
Positions of the bronchial tree on TI-EPI during 26 end-inspiration phase gating fractions of patient 1 (left). Similarly, the positions on TI-EPI of both fiducial markers in patient 2 during 17 end-expiration gating fractions are shown (right).

For patient 2, reproducibility of both clips during RGRT improved by a factor 5 (1.5 and 1.4 mm, respectively) relative to motion in all phases of 4DCT. The daily-gated positions of internal structures, after setup alignments were excluded, are summarized in figure 6. Cine-loop displays of all available TI-EPI portals of both patients are shown (see Additional files 1 and 2). On both cine-loop displays, the MLC position of treatment fields are shown at different locations during each fraction, a finding highlighting daily patient setup errors despite use of a setup protocol.

## Discussion

A surrogate structure such as the chest wall or diaphragm movement is commonly used to signal tumor position during RGRT delivery. An inability to directly observe the tumor during treatment can lead to uncertainties about the phase relationship between surrogates and the tumor or other anatomy [1]. In order to minimize the risk of internal/external correlation due to changes in breathing, our patients undergo phase RGRT delivery with audio-coaching [33]. Frequent imaging of the surrogate organ (or target, where visible) throughout treatment is essential to measure inter-fractional variations [34-36]. In the present study, we describe the use of TI-EPI for this purpose.

As a first step, we validated use of TI-EPI for verifying target positions during RGRT using a mobile phantom, and found that integral grey values on TI-EPI portals correlated well with mean object position in expiration, inspiration and during free breathing. Initial patient data also appears promising, particularly when confounding setup errors were removed by bony alignment of TI-EPIs with DRR's. The high reproducibility of the bronchial tree within the tumor region suggests that no fiducial markers may be required for the thorax in selected patients.

Our TI-EPI procedure differs from the cine EPI acquisition procedure described by Berbeco *et al*. which generated 1.6 sec-based portals every 2.1 seconds [10]. Our TI-EPI procedure involves continuously collection of image frames with short acquisition times of ~0.1 sec per image. This cine acquisition procedure allows for analysis of intra-fraction motion as portals are separately stored, but the configuration of our EPID does not support storage of separate images. Instead, the integral image (or composite EPI) obtained using our approach visualizes the overall mean position of treated anatomy during short (1.0–1.5 sec) beam-on periods for that field. Bony alignment TI-EPIs also allows for evaluation of the accuracy of RPM-triggered gating.

Not all internal structures appear to be suitable for use as internal surrogates. Ford *et al. *used fluoroscopic movement of the diaphragm as a surrogate and reported a reduction in variability of the diaphragm from 7.0 mm to 2.8 mm [37]. A similar study by Mageras *et al. *observed a reduction in diaphragm motion from 1.4 cm to 0.3 cm while gating in fluoroscopy acquisition using external fiducials [38]. Movements of the diaphragm may correlate with vertical displacement of the abdomen, but the non-rigid lung tissue may move and deform differently with respiration. In contrast, we were able to study structures with the GTV, namely the bronchial tree and fiducials.

Similarly, TI-EPIs of marker clips in a hepatic tumor also showed high daily reproducibility of < 1.6 mm during RGRT. An analysis residual fiducial motion in eight patients with lung cancer undergoing simulated gating ranged from 0.17 to 6.2 mm for different duty cycles [10]. We are currently studying TI-EPIs in a larger cohort of lung patients undergoing RGRT in order to obtain representative data. Another limitation is the relatively poor image quality of megavolt EPIs, which required us to use image enhancing tools. The pixel size of the EPI image was at best 0.78 mm, which limits the accuracy of fiducial location. In future, errors in fiducial location could be reduced with an automatic fiducial location algorithm. Another limitation of our analysis is inter-observer variation but all the TI-EPI portal matches, and the identification of internal structures were performed by a single observer (J.vSdK).

Patient setup errors also displace the tumor from its intended position but setup errors in our 2 patients appeared to be within an acceptable range. However, setup uncertainties similar to, or greater than, residual gated motion were observed for RGRT using fiducials with systematic and random errors ranging from 4 to 6 mm [18]. Improved imaging techniques are the subject of active research, and we plan to use our cone-beam CT in order to perform RPM-triggered kV radiographs before, and during, treatment.

In conclusion, RPM-based gated treatment delivery appears to be a promising technique for verifying RGRT during coached respiration. However, additional clinical study is required to confirm these findings. We plan to optimize the procedure for performing TI-EPI and are developing an on-line verification procedure prior to the start of RGRT.

## Competing interests

1. The VU University medical center has research collaborations with Varian Medical Systems (Palo Alto, CA) and GE Healthcare (Waukesha, WI) in the field of 4DCT scanning and respiration-gated radiotherapy.

2. S. Senan and F.J. Lagerwaard have received speaker's fees from GE Healthcare.

## Authors' contributions

J.vSdK., S.S. and J.C. designed the study, analysed the data and prepared the final version of the manuscript. F.dG. designed the mobility phantom and performed with J.vSdK. and J.C. all the phantom measurements. F.L. analysed the study data and prepared the final manuscript, and B.S. was involved in study design and drafting of the manuscript.

## Supplementary Material

Additional file 1 Cine-loop showing 26 TI-EPI portals acquired during gating in patient 1. The daily position of the bronchial tree is shown by the white contour, as is the planned position of the bronchial tree on DRR (in black contour). The projected grid size is 1 cm. Click here for file

Additional file 2 Cine-loop showing 17 TI-EPI portals of patient 2. Each image has two small black dots indicating the planned center position of clips (on DRR). The mean reproduced position of the clips is indicated by the white dots, and the projected grid size is 1 cm. Click here for file
